# Evidence of RedOX Imbalance during Zika Virus Infection Promoting the Formation of Disulfide-Bond-Dependent Oligomers of the Envelope Protein

**DOI:** 10.3390/v14061131

**Published:** 2022-05-24

**Authors:** Grégorie Lebeau, Jonathan Turpin, Etienne Frumence, Daed El Safadi, Wissal Harrabi, Philippe Desprès, Pascale Krejbich-Trotot, Wildriss Viranaïcken

**Affiliations:** PIMIT, Processus Infectieux en Milieu Insulaire Tropical, Université de La Réunion, INSERM UMR 1187, CNRS 9192, IRD 249, Plateforme CYROI, 97490 Sainte-Clotilde, Ile de La Réunion, France; gregorie.lebeau@univ-reunion.fr (G.L.); jonathan.turpin@univ-reunion.fr (J.T.); etienne.frumence@univ-reunion.fr (E.F.); daedalsafadi@gmail.com (D.E.S.); wissalharrabi500@yahoo.fr (W.H.); philippe.despres@univ-reunion.fr (P.D.)

**Keywords:** Zika virus, unfolded protein response, ER stress, oligomer, disulfide bond, amyloid aggregates

## Abstract

Flaviviruses replicate in membrane factories associated with the endoplasmic reticulum (ER). Significant levels of flavivirus viral protein accumulation contribute to ER stress. As a consequence, the host cell exhibits an Unfolded Protein Response (UPR), subsequently stimulating appropriate cellular responses such as adaptation, autophagy or apoptosis. The correct redox conditions of this compartment are essential to forming native disulfide bonds in proteins. Zika virus (ZIKV) has the ability to induce persistent ER stress leading to the activation of UPR pathways. In this study, we wondered whether ZIKV affects the redox balance and consequently the oxidative protein folding in the ER. We found that ZIKV replication influences the redox state, leading to the aggregation of the viral envelope protein as amyloid-like structures in the infected cells.

## 1. Introduction

Zika virus (ZIKV) is a pathogenic single-stranded RNA virus belonging to the *Flaviviridae* family, together with the Yellow Fever virus (YFV), Dengue virus (DENV), West Nile virus (WNV) and Japanese Encephalitis virus (JEV). Among the pathogenic *flaviviruses*, ZIKV has gained notoriety in the last ten years due to several outbreaks and serious clinical concerns. Notably, neurological complications have been described, including Guillain-Barré syndrome (GBS) and congenital malformations (Congenital Zika Syndrome—CZS), prompting specific vigilance for pregnant women in the case of the ZIKV epidemic [1,2]. Otherwise, the ability of ZIKV to be transmitted sexually alongside to vector-borne transmission and the evidence of its persistence in some tissues conversely to other *flaviviruses* raised the interest in specific ZIKV–host interactions. This has led to a growing number of studies to decipher these interactions at the cellular and molecular levels. Similar to other *flaviviruses*, ZIKV replication occurs in Endoplasmic Reticulum (ER) invaginations of infected cells and induces an accumulation of viral proteins [3,4,5]. Genomic RNA translation leads to the viral polyprotein incorporation in the ER membrane, followed by its proteolytic cleavage. Translation of the polyprotein, initially enclosed through the ER membrane, results in three structural (C, prM, and E) and seven non-structural (NS1, NS2A, NS2B, NS3, NS4A, NS4B, and NS) proteins. Six of these proteins remain membrane-bound, as do the prM and E proteins that constitute the future viral envelope.

In addition to its key roles in cell physiology and homeostasis, the ER is an essential cellular component in the execution of the viral cycle. The main function of the ER is the regulation of folding and post-translational modifications of neo-synthesized proteins. Quality control of folding by the ER determines the outcome of proteins destined for centrifugal transport and the secretory pathway. Indeed, protein folding involves N-glycosylation, an oxidative environment favorable to disulfide bridge formation in the ER lumen, and the presence of several Ca^2+^-dependent molecular chaperones (e.g., calreticulin, GRP78/BiP and GRP94) [6,7]. The latter is in particular involved in the stabilization of protein folding intermediates. ER homeostasis can be disrupted by many factors such as glucose deprivation, hypoxia, calcium imbalance or protein accumulation [8]. The resulting ER stress causes cellular adaptive responses, such as the Unfolded Protein Response (UPR), which is activated in an attempt to restore ER homeostasis.

Upon virus infection, the sudden and massive influx of ER-processed viral proteins typically results in ER stress and UPR in infected cells. Such cell responses contribute to several modes of host defense, i.e., antiviral programs, immune responses [9,10] and commitment to autophagy or cell suicide [11,12]. The crosstalk between ER stress, UPR, autophagy and apoptosis is particularly important during infection. Each of these responses can affect cellular homeostasis and consequently the efficiency of viral replication and spread.

The ability of ZIKV to induce UPR in response to ER stress in several cell types has been previously studied [13,14,15,16,17,18,19]. The infection was accompanied by morphological changes in cell organelles, including remodeling of the ER into a swollen shape [20,21]. As aforementioned, during viral infection, ER stress is usually the result of an increasing number of viral proteins to be processed by the ER. This influx of proteins disrupting ER homeostasis is likely to trigger UPR and a translational shutdown which will have negative effects on the replicating virus. We have previously shown that ZIKV is able to hijack the ATF6 and CHOP pathways during UPR [18,19]. This control by ZIKV results in persistent unresolved ER stress and delayed induction of cell death, which benefit the virus and may even promote its long-term persistence in the body [22].

Data from the literature indicate that persistent ER stress is associated with a loss of glutathione activity, which, in turn, provokes an imbalance in redox homeostasis [23]. Indeed, the ER provides a relatively oxidizing thiol-disulfide environment suitable for the oxidative folding of co-translationally translocated proteins. Correct folding of proteins is in part dependent on disulfide bond formation, a reversible process that is achieved by a thiol-disulfide exchange reaction [24]. This exchange is mediated by a catalytic redox cycle, which involves ERO-1 (ER oxidoreductin 1), PDI (Protein Disulfide Isomerase) and GSH (reduced glutathione), to ensure the formation and isomerization of disulfide bonds in proteins [25]. The stability of the thiol-disulfide system in the ER relies on the glutathione redox pair, which acts as a powerful guardian of the redox balance. Thus, the ratio of the glutathione reduced form (GSH) to the oxidized glutathione disulfide form (GSSG) in the ER is an indicator of the redox homeostasis [26]. It should be noted that the specific redox conditions in the ER allow the formation of disulfide bonds within the neo-synthesized polypeptides. These covalent bonds are necessary for the functional three-dimensional configurations of proteins in progress in the ER. Due to the importance of an oxidative environment for the correct folding of proteins [27], a redox imbalance could be a trigger for protein misfolding and the formation of non-native disulfide bonds during ER stress [28].

We, therefore, wondered whether, during ZIKV infection, persistent ER stress might be related to an imbalance of the redox status. We questioned to what extent this would contribute to the accumulation of misfolded proteins. Under infection conditions, we examined the GSH/GSSG balance and showed that it significantly decreased, subsequently impairing the redox balance. We showed that in ZIKV-infected cells, the envelope protein (ZIKV-E) could oligomerize in large molecular weight complexes with insoluble characteristics. The presence and accumulation of such oligomers seem to correlate with the decrease in the redox potential of glutathione in infected cells. The redox imbalance generated during infection could be responsible for the generation of amyloid-like protein structures linked by inappropriate disulfide bonds. This hypothesis would be consistent with fluorescence microscopy observations of thioflavin T labeling, which reveal the presence of protein aggregates that colocalize with the ZIKV-E protein. All of these data suggest a change in oxidative protein folding in the ER due to persistent ER stress and ER redox imbalance during ZIKV infection. Misfolded proteins would cause persistent stress, promoting a spiral in which more inappropriate disulfide bonds would form, promoting protein aggregation.

## 2. Materials and Methods

### 2.1. Cells, Virus and Reagents

The clinical isolate PF-25013-18 (PF13) of ZIKV [29] was used for all infections. A549 cells (ATCC, CCL-185) and HEK-293T cells (ATCC, CRL-3216) were cultured at 37 °C under a 5% CO_2_ atmosphere in Dulbecco’s modified eagle medium (P04-3500, PAN-Biotech™) supplemented with 10% heat-inactivated fetal bovine serum (FBS, P40-37500, PAN-Biotech™). Briefly, A549 cells were infected for 48 h with MOI ranging from 0.5 to 5. HEK-29T cells were transfected with 2.5 µg of plasmid encoding ZIKV prM/E from epidemic BeH819015 (BR15) isolated in Brazil in 2015.

Immunodetection of the viral proteins was performed using the mouse anti-pan flavivirus E protein 4G2 (RD Biotech^®^, Besançon, France) or with the rabbit anti-EDIII ZIKV, which was described previously [30]. Donkey anti-mouse Alexa Fluor 488 and anti-rabbit Alexa Fluor 594 IgG antibodies were purchased from Invitrogen (AB_141607 and AB_2556547, Thermofisher^®^, Les Ulis, France). Horseradish peroxidase-conjugated anti-rabbit (ab97051) and anti-mouse (ab6789) antibodies were from Abcam^®^ (Cambridge, UK). Thioflavin T, a benzothiazole dye that increases in fluorescence upon binding to amyloid fibrils and protein aggregates, was purchased from Sigma-Aldrich^®^ (243.003516.10, Humeau, La Chapelle-Sur-Erdre, France). GSH/GSSG-Glo™ assay was purchased from Promega (V6611, Charbonnières-les-Bains, France). Tris (2-carboxyethyl)phosphine (TCEP) is a reducing reagent used at 5 µM and purchased from Sigma-Aldrich^®^ (243.075259.02, Humeau, La Chapelle-Sur-Erdre, France). Transfection was achieved using Lipofectamine 3000^®^ (L3000-015, Invitrogen, Carlsbad, CA, USA) according to the manufacturer’s recommendations.

### 2.2. Cell Fractionation and Western Blot

For fractionation, cells were washed with PBS and lysed at the concentration of 1 × 10^4^ cells·μL^−1^ in buffer A (0.2% Triton X-100, 50 mM Tris-HCl pH 7.5, 150 mM NaCl, 2.5 mM EDTA) as before [31]. Following lysis, the insoluble fraction was separated by centrifugation at 3400× *g* for 10 min. Pellets were enriched in non-folded proteins, and the supernatant was enriched in soluble proteins. All fractions were used in corresponding Western blots.

For other Western blot assays, samples were either lysed with TEN buffer (0.1 M Tris-Cl pH 8.0, 0.01 M EDTA pH 9.0 and 1 M NaCl) or RIPA buffer. Cell lysates were then sonicated, treated in Laemmli buffer with or without DTT/TCEP and heat-treated at 95 °C for 5 min. Finally, samples were processed by SDS-PAGE, transferred onto nitrocellulose membrane as previously reported [32], and subsequently incubated with the aforementioned antibodies at the following dilutions: 1:1000 for 4G2, rabbit anti-EDIII and β-actin, 1:2000 for HRP-conjugated anti-rabbit and anti-mouse.

### 2.3. Immunofluorescence and Thioflavin T Staining

A549 cells were grown, infected or treated on glass coverslips. They were further fixed with 3.7% formaldehyde at room temperature for 10 min. Fixed cells were permeabilized with 0.1% Triton X-100 in PBS for 5 min. Coverslips were incubated with primary antibodies (1:1000 dilution) in 1× PBS 1% BSA for two hours and with Alexa Fluor-conjugated secondary antibodies (1:1000, Invitrogen) for 1h. Nucleus morphology was revealed by DAPI staining. According to Beriault and Werstuck [33], after immunodetection of ZIKV EDIII and DAPI staining, amyloid indicator thioflavin-T was added to the coverslips at 5 µM for 10 min before proceeding to mount. The coverslips were mounted with VECTASHIELD^®^ (Clinisciences, Nanterre, France), and fluorescence was observed using a Nikon Eclipse E2000-U microscope. Images were captured and processed using a Hamamatsu ORCA2 ER camera and the imaging software NIS-Element AR (Nikon, Tokyo, Japan).

### 2.4. Glutathione Abundance Measurement

A549 cells were either infected or not with PF13 at MOI 5. After 48h of infection, the antioxidant capacity of glutathione was assessed in infected cells versus mock infected cells using the luminescence-based GSH/GSSG-Glo™ assay kit following the manufacturer’s recommendations. Briefly, after treatment, the cell culture supernatant was removed, and cells were lysed either with Total Glutathione Lysis Reagent or Oxidized Glutathione Lysis Reagent. After 5 min of agitation, Luciferase Generation reagent was added. Thirty minutes after incubation at RT, Luciferin Detection Reagent was added. Luminescence emitted was measured after brief agitation and 15 min of incubation using a microplate reader (Tecan FLUOstar^®^ Omega). The GSH/GSSG ratio for a sample was calculated using the following formula:

$$\frac{Net\;total\;glutathione\;luminescene\;-\;Net\;oxidized\;glutathione\;luminescene}{Net\;oxidized\;glutathione\;luminescene/2}$$

### 2.5. Protein Sequence Alignment

Flavivirus prM/E proteins were aligned using Clustal Omega. Following sequences were used: POLG_YEFVA—Yellow Fever virus, POLG_DEN2N—Dengue virus, ARB08102.1—Zika virus PF13.

### 2.6. Statistical Analyses

All values are expressed as mean ± SD of at least three independent experiments. After normality tests, comparisons between infected and mock infected cells were analyzed using a *t*-test. Values of *p* < 0.05 were considered statistically significant. All statistical tests were conducted using Graph-Pad Prism version 9 software (GraphPad Software, San Diego, CA, USA, Available online: http://www.graphpad.xn--com-9o0a).

## 3. Results 

### 3.1. Glutathione Abundance in the Course of ZIKV Infection

**Figure 1:**
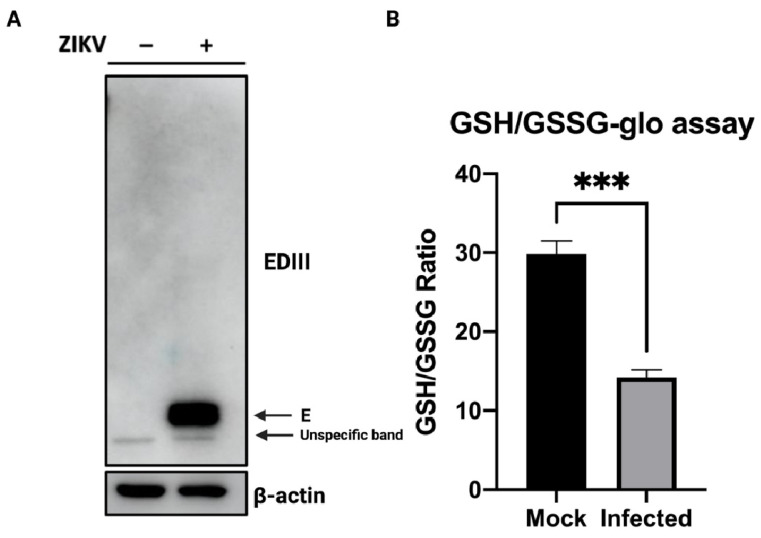
ZIKV infection is associated with redox imbalance. (**A**) ZIKV infection of A549 cells. A549 cells were either mock infected or infected with ZIKV at MOI 5 for 48 h. Cell extracts were reduced using DTT and heat-treated at 95 °C for 5 min. Western blot analysis of cell extracts following infection leads to detection of ZIKV envelope protein (~55 kDa). ZIKV-E protein is detected using rabbit anti-EDIII antibody. This is representative of three independent experiments. (**B**) ZIKV leads to a decrease in the GSH/GSSG ratio, indicating a loss of glutathione activity in infected A549 cells. 48 h.p.i. glutathione activity was assessed in mock infected vs. infected cells. ***: *p* < 0.01. h.p.i: h post-infection.

We and others have reported that ZIKV infection, like many other enveloped viruses, can trigger ER stress and UPR in the host cell [13,14,15,16,17,18,19]. It is still unknown whether a viral factor is more specifically involved in ZIKV-mediated ER stress. Disruption of ER homeostasis may be followed by an altered redox state and may be associated with impaired glutathione availability and sulfur tripeptide metabolism [23,34]. We wondered whether ZIKV-mediated ER stress could be related to a similar phenomenon. Therefore, we infected A549 cells with ZIKV at MOI 5 for 48h. As shown in Figure 1A, Western blot analysis (in reducing conditions) of cell extracts harvested 48 h post-infection (h.p.i.) shows ZIKV envelope protein (~55 kDa), indicating infection and viral replication in A549 cells. At the same time, infected and mock infected A549 cells were lysed to compare the relative level of oxidized and reduced glutathione under these two conditions. Interestingly, following infection, an approximately three-fold decrease in the GSH/GSSG ratio is observed in infected cells (Figure 1B). The decrease in glutathione reduced form observed presumably relates to a loss of glutathione activity during ZIKV infection.

### 3.2. Detection of Amyloid-like Structures in ZIKV-Infected Cells

The imbalance in the GSH/GSSG ratio suggests an alteration in the redox state during ZIKV infection. Redox imbalance is known to participate in ER stress [35], which has to be resolved by UPR. However, it has been shown that during ZIKV infection, incomplete UPR results in persistent and poorly resolved ER stress [18,19]. Under these conditions of persistent stress and redox imbalance, the accumulation of oxidized protein could lead to the formation and the aggregation of amyloid-like structures [35].

**Figure 2:**
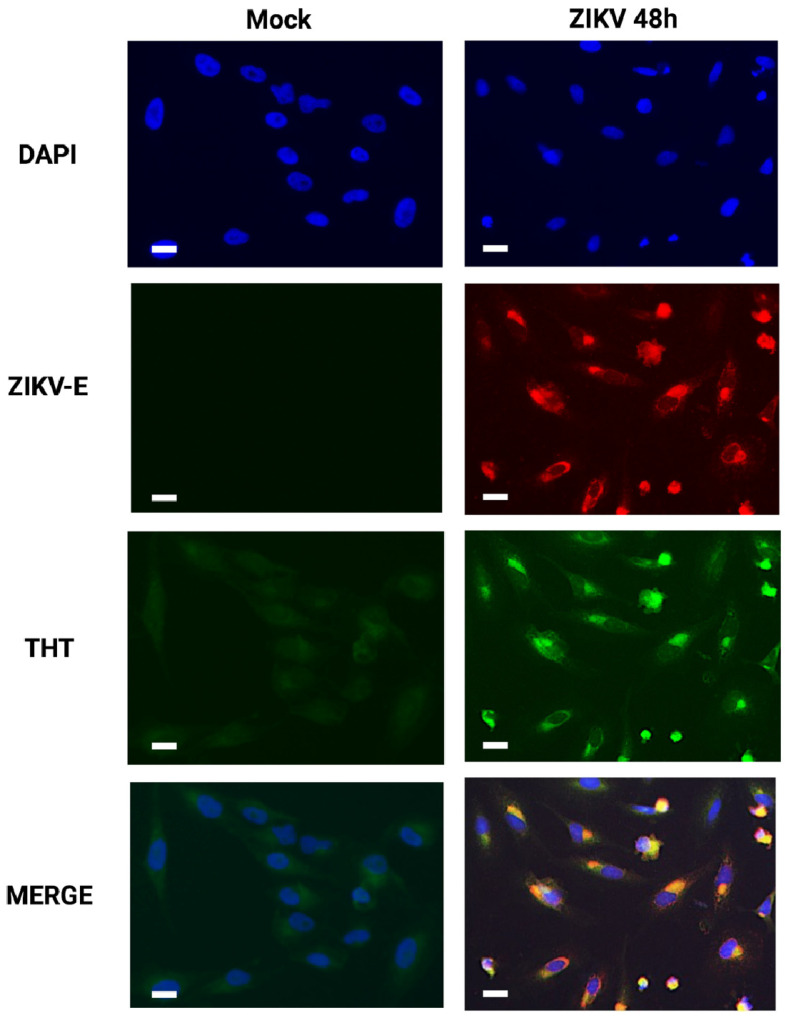
Amyloid-like protein accumulation during ZIKV infection. A549 cells infected with ZIKV at MOI 1 for 48 h were incubated with Thioflavin-T (THT), a green fluorescent ER stress indicator that binds aggregated proteins. Immunodetection of ZIKV envelope protein confirmed the infection of the cells. Scale bar: 5 µm. All images are representative of three independent experiments.

To assess such a hypothesis, we infected A549 cells with ZIKV at MOI 1 for 48 h. Cells were fixed and examined by immunofluorescence. Intracellular virus replication was examined using a monospecific anti-E polyclonal antibody, and fluorescent dye Thioflavin-T (THT) was used to detect protein aggregates and any form of amyloid-like structures [33]. At 48 h.p.i., only A549 cells positively stained for the E protein showed a positive signal for THT as compared to mock-infected cells, suggesting that amyloid-like protein aggregates can occur in ZIKV-infected cells (Figure 2). Interestingly, the staining of amyloid-like structures and the staining of the ZIKV-E protein overlap (Figure 2). We have previously demonstrated that ZIKV-E localizes to the ER by co-immunodetection of calnexin [18], and it is validated that E protein is processed and folded in the ER before the assembly of viral particles in the secretory pathway [36]. Thus, the colocalization of ZIKV-E and THT labeling suggests an accumulation of amyloid-like structures in the stressed ER of infected cells. Furthermore, these amyloid-like structures may be related to misfolding of the viral envelope protein.

### 3.3. Detection of Disulfide-Bonded E Oligomers in ZIKV-Infected Cells

**Figure 3:**
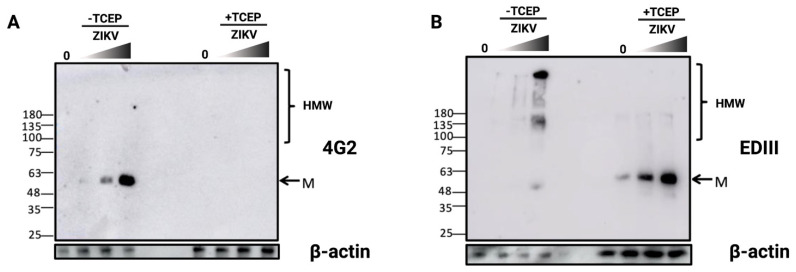
ZIKV infection results in the formation of disulfide-crosslinked E oligomers. (**A**) ZIKV-E in native conformation contains intermolecular disulfide bridges. After infection of A549 cells with ZIKV in a dose-dependent manner (MOI 0.5, 1 and 5), protein extracts were either treated or not with TCEP before SDS-PAGE. Western blot analysis was performed with mouse 4G2 antibody, which recognizes a conformational epitope of the E protein. (**B**) ZIKV-E protein forms disulfide-cross-linked oligomers. A549 cells were infected with increased quantities of ZIKV (MOI 0, 0.5, 1 and 5), and the total fraction was treated or not with TCEP before Western blotting. Western blot assay was achieved with a rabbit anti-EDIII antibody. M indicates the 55 kDa monomer, and HMW indicates high molecular weight oligomers of ZIKV-E. These images are representative of three independent experiments.

To determine whether a relationship exists between the expression of the E protein and the formation of amyloid-like structures in infected cells, A549 cells were infected with increasing inputs of ZIKV (Figure 3). Protein extracts were collected 48 h.p.i. and either reduced or not using Tris (2-carboxyethyl) phosphine (TCEP) as a reducing agent. Protein samples were then analyzed by immunoblot assay (Figure 3A). Under non-reducing conditions, we can observe a single band when we use the 4G2, an anti-pan-flavivirus envelope protein antibody. The signal corresponds to the monomeric form of ZIKV-E protein (M, ~55 kDa), and it increases in an MOI-dependent manner (Figure 3A). Interestingly, this band is no longer observed when using TCEP (Figure 3A). The lack of recognition of ZIKV-E protein under reducing conditions is consistent with literature data indicating that the 4G2 antibody binds a conserved conformational epitope overlapping the E fusion loop [37]. This suggests that oxidized disulfide bonds may stabilize the tertiary structure of ZIKV-E protein and be necessary for conformational epitope recognition.

Immunoblotting was repeated in the same conditions as described above but this time using an anti-EDIII antibody. Under non-reducing conditions, the monomeric form of ZIKV-E protein was not predominantly detected. In contrast, high molecular weight (HMW) signals of ZIKV-E protein are detected. These forms of over 100 kDa in size could correspond to oligomers of the E protein (HMW, Figure 3B). Conversely, after TCEP treatment and immunodetection with anti-EDIII antibody, the monomeric form of ZIKV-E (M, Figure 3B) prevails, and the oligomeric forms are no longer detected. These observations suggest that during ZIKV infection, E proteins are produced and accumulated in different forms, including high molecular weight forms that are not detectable when reduced. These oligomeric forms would therefore be stabilized by disulfide bonds. To confirm the possible formation of envelope protein oligomers, we decided to examine the electrophoretic migration patterns under reducing and non-reducing conditions after the overexpression of ZIKV prM/E in HEK-293T cells. Interestingly, the same HMW forms of the E protein were observed under non-reducing conditions (Figure S1).

During infection, the envelope protein of flavivirus is mainly folded in the ER, and its monomeric tertiary structure depends on the generation of intramolecular disulfide bonds that occur in this cellular compartment [38]. Oligomeric forms resulting from weak interactions between the individual monomers have also been described [39]. To the best of our knowledge, we are the first to demonstrate the possibility of E-oligomers formation in the context of ZIKV infection due to disulfide covalent bond formation.

**Figure 4:**
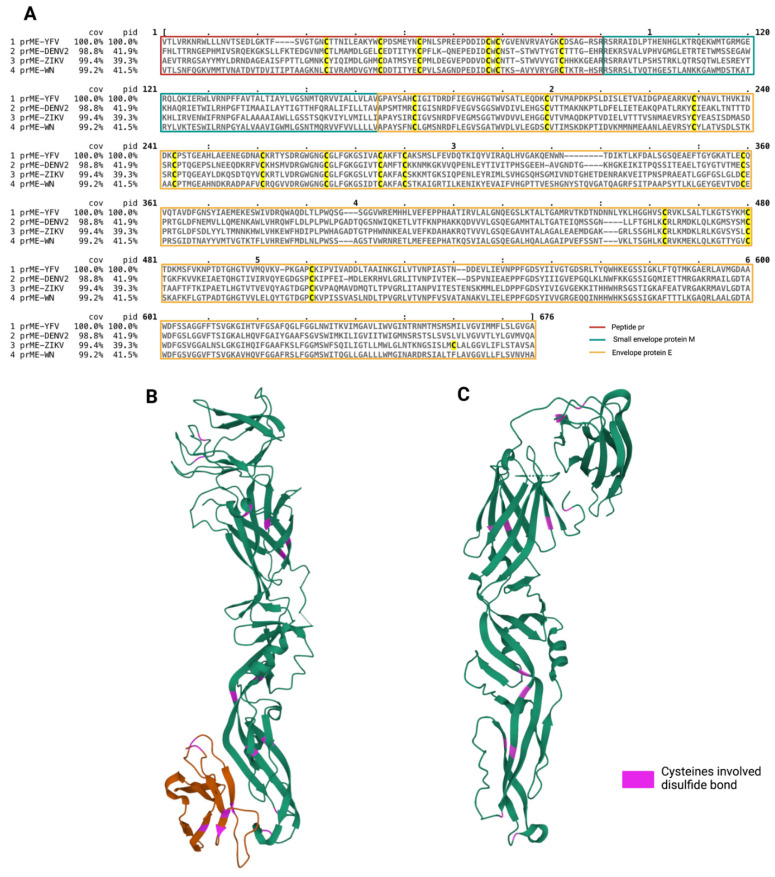
Disulfide bonds in *flavivirus* prM/E protein. (**A**) Alignment of prM/E protein sequence from YFV (POLG_YEFVA), DENV2 (POLG_DEN2N), WNV (POLG_WNV9) and ZIKV (ARB08102.1). The cysteine positions are highlighted in yellow. (**B**) Crystallography structure of DENV2 prM/E (3C6E), with cysteine positions involved in disulfide bonds formation highlighted in pink. (**C**) Crystallography structure of ZIKV-E (5JHM), with cysteine positions involved in disulfide bonds formation highlighted in pink.

Thus, we wondered whether this ability was a specificity of the ZIKV-E protein among the different flaviviral envelope proteins. We therefore aligned the prM/E amino acid sequences of YFV, DENV, WNV and ZIKV, as shown in Figure 4A. All *flaviviruses* assessed have highly conserved cysteines with six residues belonging to the prM protein and twelve to the E protein. Interestingly, ZIKV-E contains a 13th cysteine residue. As illustrated in Figure 4B for DENV2 or in the literature for WNV, the 12 well-conserved cysteine residues within E protein are all adjacent and involved in disulfide proximity bonds [38,40]. This feature is also found in ZIKV prM/E (Figure 4C), where 12 of the 13 cysteine residues in E are highlighted and form disulfide bonds through their adjacent positions, the thirteenth residue not being accessible for an oxidized bond formation in the native ZIKV-E form. The monomer of ZIKV-E would then be stabilized for its proper folding by 6 disulfide bonds, as are the E proteins of other *flaviviruses*. In contrast, the presence of an additional cysteine specific to ZIKV-E could account for intermolecular disulfide bond formation when the protein incorporated into the stressed ER is misfolded. Here, we propose that the oligomerization of E proteins is the result of misfolding and abnormal conformations due to the formation of the wrong intra- and/or intermolecular disulfide bonds between polypeptides. We hypothesize that persistent ER stress could be a potent initiator of this phenomenon and that the additional cysteine plays a role in increasing the combinatorial possibilities for the formation of these non-native bonds between the cysteine residues implicated in disulfide bonds.

### 3.4. ZIKV E Protein Forms Insoluble Oligomers during Infection

**Figure 5:**
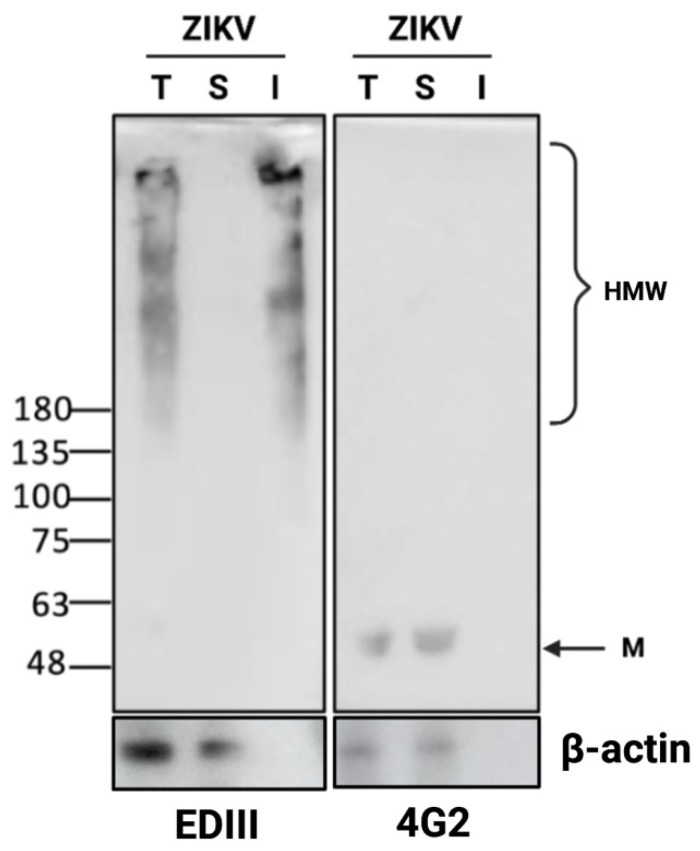
ZIKV E disulfide-cross linked oligomers are insoluble. A549 cells were infected with ZIKV at MOI 5. 48 h.p.i. cells were lysed in TX100 buffer A and fractionated by centrifugation at 3400× *g*. An equal volume of total (T), soluble (S) and insoluble (I) fractions were separated under non-reducing conditions and immuno-blotted for ZIKV-E with anti-EDIII or 4G2 antibodies. M indicates the 55 kDa monomer, and HMW indicates high molecular weight oligomers of ZIKV-E. This is representative of three independent experiments.

The oligomerization of proteins due to the cross-linking of non-native disulfide bonds may lead to the formation of protein aggregates, one hallmark of which will be a loss of solubility [41]. As previously stated, we have been able to show that ZIKV induces unresolved ER stress in infected cells that have a prolonged lifespan [18,19]. These conditions, which are combined with the redox imbalance (Figure 1), are likely to influence ER function and protein folding. Indeed, during infection, a large amount of misfolded ZIKV-E protein accumulates in the form of oligomers. In agreement with the results obtained with the THT-amyloid-like imaging (Figure 2), the detection of E-protein oligomers (Figure 3) could be consistent with the formation of insoluble aggregates. To test this hypothesis, we prepared protein extracts from ZIKV infected cells using a buffer that contains a detergent able to solubilize integral membrane proteins. Protein extracts were then fractionated according to their solubility by low-speed centrifugation (3400× *g*) to prevent microsome sedimentation. Total protein extract, soluble fraction and insoluble fraction were then used for Western blot analysis. Immunoblot using 4G2 antibody shows a unique band at ~55 kDa in both the total protein extract and the soluble fraction, corresponding to the monomeric form of the E protein (Figure 5). The anti-EDIII antibody detected the E oligomers in the insoluble fraction (Figure 5), suggesting that ZIKV envelope protein can be engaged in the formation of insoluble aggregates. These latest results confirm that ZIKV infection leads to an accumulation of high molecular weight oligomers of the E protein, in which misfolded polypeptides are associated with each other by disulfide bonds, thus resulting in protein aggregation with insoluble and amyloid features.

## 4. Discussion

**Figure 6:**
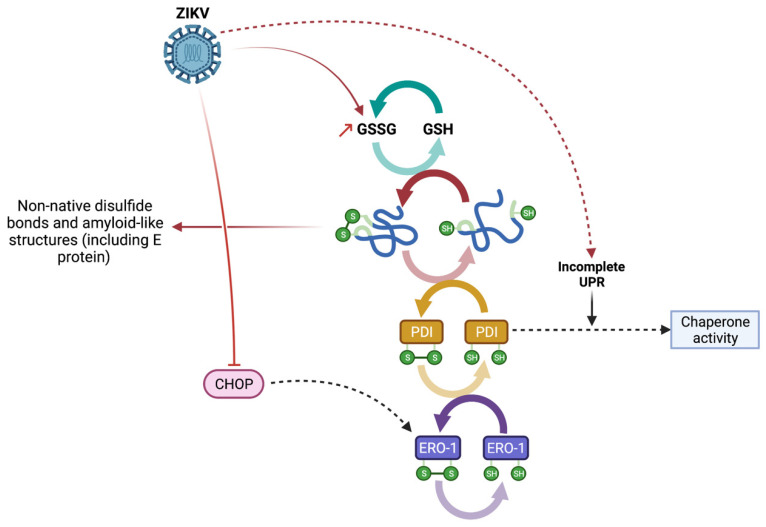
Graphical overview of the impaired redox cycle and the formation of protein aggregates during ZIKV infection.

The shaping of proteins that transit in the ER is a crucial step in their maturation, necessary for them to adopt a native conformation that will condition their future biological functions. This 3D configuration relies on the correct folding of polypeptide chains and involves both weak interactions and the formation of specific covalent bonds between cysteine residues. Disulfide bond formation is a reversible process that is achieved by a thiol-disulfide exchange reaction [24]. This exchange is permitted by a catalytic redox cycle involving ERO-1, PDI and GSH. This phenomenon is ensuring the formation and isomerization of disulfide bonds in proteins [25] (Figure 6). This oxidative protein folding occurs in an oxidized environment to maintain native protein conformation. However, a significant decrease in GSH/GSSG ratio is known to lead to protein misfolding and inappropriate formation of disulfide bonds. In eukaryotic cells, GSH is the most abundant non-protein thiol [42], which is oxidized to glutathione disulfide (GSSG) when it assists in disulfide-bond reduction (Figure 6). Upon ER stress and UPR, ER environment becomes increasingly reduced due to the consumption of GSH [43]. Thus, the ratio change observed during ZIKV infection (Figure 1) can be related to prolonged UPR and unresolved ER stress. During ER stress, ERO-1 and PDI are induced through UPR to favor protein folding as an adaptive response. The induction of ERO-1 during UPR involves the transcription factor CHOP [44]. However, CHOP expression and activity are downregulated during ZIKV infection [19], thus probably affecting ERO-1 expression (Figure 6). In addition, homeostasis impairment imposed by the UPR, such as during ZIKV infection [18,19], is known to be associated with a compromised ER protein oxidation and a PDI chaperone activity rather than a disulfide isomerase activity for PDI [45]. All of these points (ERO-1 downregulation, PDI activity switch and decreased GSH) illustrate that during ZIKV infection, the redox state is affected in relation to a decreased thiol-disulfide exchange reaction (Figure 6). Accordingly, protein misfolding and the formation of non-native disulfide bonds during ZIKV infection can occur. Such changes in ER homeostasis and activities are likely to lead to the formation of aggregates and amyloid-like structures, as shown with THT staining in ER upon ZIKV infection (Figure 2). 

During the infection process, viral proteins incorporated into the ER are subject to the same folding and control rules, and the organized formation of disulfide bridges contributes to their functional shaping. ZIKV-E contains cysteine residues that allow the formation of disulfide bonds important for the protein’s native conformation. Using the monoclonal pan-flavivirus 4G2 antibody, we were then able to detect a conformational epitope in the monomeric form of the E protein (Figure 3A). Conversely, after treatment with a reducing agent, the epitope became undetectable with the 4G2 antibody. This confirmed that intramolecular disulfide bridges shape the native E protein. These observations are in agreement with data on other *Flavivirus* (e.g., WNV, DENV), indicating that all cysteine in the E protein is engaged in intramolecular disulfide bonds [38,40]. Unexpectedly, the use of a monospecific antibody raised against the antigenic domain III of the E protein (EDIII) allowed us to reveal that the E protein exists in several forms during ZIKV-infection. One is the native monomeric form with intrachain disulfide bonds, and the others are high molecular weight oligomers with unfitted intermolecular disulfide bonds (Figure 3B). The formation of such E-related structures can be related to a change in redox state associated with a thiol-disulfide exchange reaction reduction in link with a persistent ER stress, as described earlier. Sequence analysis of prM/E for cysteine content highlights the presence of an additional cysteine in ZIKV-E compared to other *Flavivirus* (Figure 4). This free cysteine could be related to a greater susceptibility of ZIKV-E protein to form non-native disulfide-bonded oligomers compared to the other *Flavivirus* in the case of redox imbalance and misfolding. Finally, the non-native disulfide-bonded oligomers of E remain in an insoluble fraction (Figure 5), which is consistent with protein aggregation. As THT staining co-localized with ZIKV-E protein (Figure 2), we can therefore argue that these aggregates of ZIKV-E form amyloid-like structures. 

The next challenge will be to decipher the pathophysiological impact of these amyloid-like structures during ZIKV infection, especially in microcephaly and other neurological disorders, as these structures are known triggers of apoptosis in the nervous system [41,46]. The consequence of the co-existence of ZIKV-E with intramolecular disulfide bonds and oligomer of E with non-native intermolecular disulfide bonds for virus cycle and particularly for viral egress should be also eluded.

## Data Availability

The data presented in this study are available on request from the corresponding author.

## Supplementary Materials

The following supporting information can be downloaded at: https://www.mdpi.com/article/10.3390/v14061131/s1, Figure S1: ZIKV prM/E overexpression results in the formation of disulfide-crosslinked oligomers.

## Abbreviations

CHOP	C/EBP Homologous Protein
DENV	Dengue Virus
EDIII	Domain III of E protein
ER	Endoplasmic Reticulum
ERO-1	ER oxidoreductin 1
GSH	Reduced glutathione
GSSG	Oxidized glutathione
PDI	Protein Disulfide Isomerase
TCEP	Tris (2-carboxyethyl)phosphine
THT	Thioflavin T
UPR	Unfolded Protein Response
WNV	West Nile Virus
YFV	Yellow
ZIKV	Zika Virus
ZIKV-E	Zika Virus envelope protein
