# Supplementary material for: Evidence of RedOX Imbalance during Zika Virus Infection Promoting the Formation of Disulfide-Bond-Dependent Oligomers of the Envelope Protein
Source: Viruses. 2022 May 24;14(6):1131. doi: 10.3390/v14061131 (PMC9227265; doi:10.3390/v14061131)
Supplement: Supplementary file 1 [file viruses-14-01131-s001.zip › viruses-1684905-supplementary.pdf]

viruses MDPI

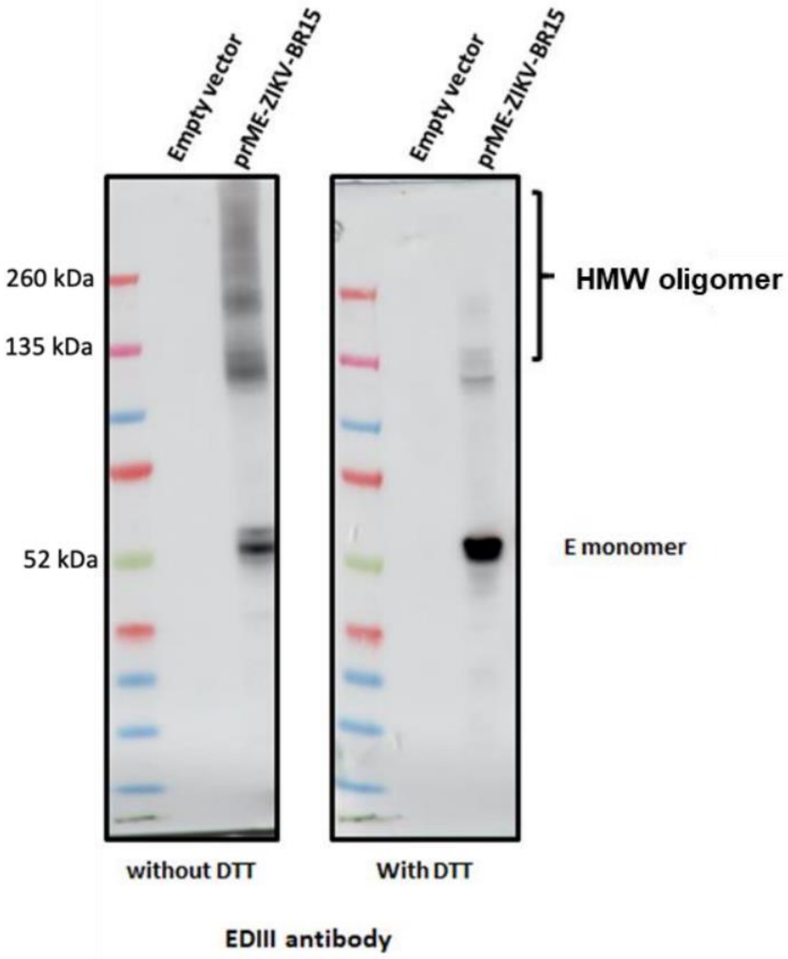

Figure S1. ZIKV prM/E overexpression results in the formation of disulfide-crosslinked oligomers. HEK-293T cells were transfected with a plasmid encoding ZIKV prM/E protein. After 24h, protein extracts were harvested and either treated or not with DTT before SDS-PAGE. Western blot assay was achieved with a rabbit anti-EDIII antibody. These images are representative of three independent experiments.
